# Pearl-necklace nano-chains from tomato peel lycopene carbon dots: Enhanced Colloidal stability, pH sensing, and antibacterial monitoring

**DOI:** 10.1186/s11671-026-04911-0

**Published:** 2026-10-08

**Authors:** Hebat-Allah S. Tohamy

**Affiliations:** Cellulose and Paper Department, National Research Centre, 33 El Bohouth Str, P.O. 12622, Dokki Giza, Egypt

**Keywords:** Carbon dot nanosheets, Pearl-necklace nano-chains, Lycopene, Tomato peel wastes, Carboxymethyl carbon dots, Microwave-assisted Synthesis, Fluorescent nano-probes, Surface functionalization, Pathogen detection

## Abstract

Transforming industrial tomato peel waste (a dense matrix of lignocellulosic fibers and bioactive lycopene) into advanced protective functional nano-probes. Utilizing a one-pot microwave-assisted hydrothermal route to synthesize carbon dot-decorated nanosheets (N-TP-CDs), which were then evolved into interconnected 1D pearl-necklace nano-chains (PN-NCs) via strategic carboxymethylation (Mercerization Depth = 0.53). Transmission Electron Microscopy (TEM) confirmed a morphological shift to 1D networks of pearls (2.88–4.39 nm), while Density Functional Theory (DFT) calculations revealed a narrowed energy gap (E_g_ = 3.3170 eV) and an increased dipole moment (36.39 Debye) for enhanced electronic sensitivity. Demonstrated superior colloidal stability, a naked-eye chromogenic response to pH fluctuations, dual-mode selective NIR fluorescence shifts, and near-total antimicrobial efficacy (99.99%±1% against *S. aureus* and 99.62 ± 2% against *E. coli*). By merging real-time diagnostic visibility with a potent bacterial inhibition efficiency, this research offers a sustainable, high-performance tool for intelligent food packaging and environmental monitoring.

**Figure:**
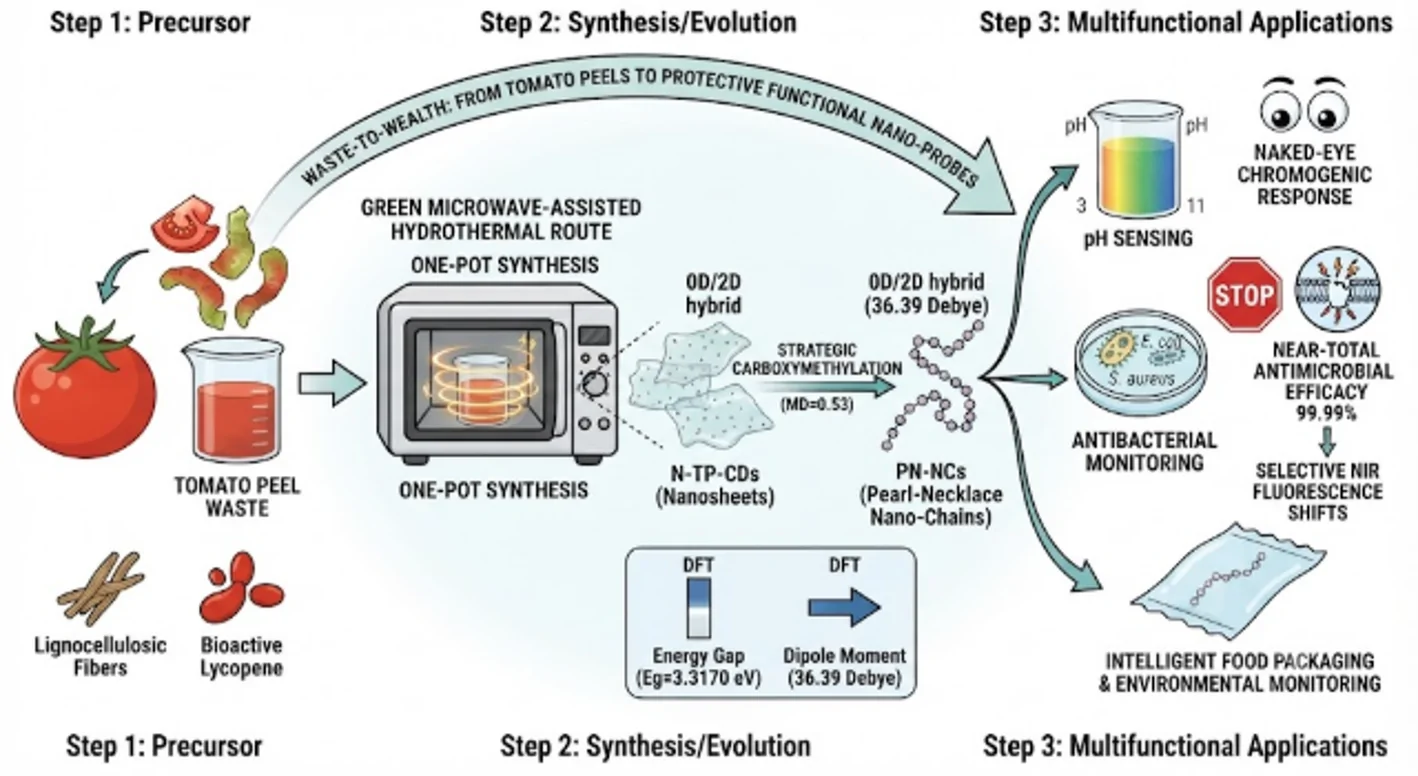

## Introduction

Across the globe, the industrial processing of tomatoes leaves behind a staggering legacy of environmental neglect, with millions of tons of skins and peels discarded annually as low-value agro-industrial residue [1–6]. However, viewing these wastes merely as a disposal burden overlooks a sophisticated chemical treasure trove; beneath their crude exterior, tomato peels are a dense matrix of lignocellulosic fibers and bioactive lycopene, serving as a naturally pre-functionalized precursor for advanced nanotechnology [7–11]. This research seeks to shift the narrative from simple waste management to a high-precision waste-to-wealth hierarchy, where the complex molecular architecture of the peel is not destroyed, but strategically evolved. By employing green, microwave-assisted hydrothermal chemistry, we can bypass traditional, harsh disassembly methods to achieve a rapid and energy-efficient transformation. This process treats the biomass as a refined template, precisely unzipping its organic framework to nucleate ultra-small carbon dots that remain anchored to their parent nanosheet scaffolds [12–14].

The remarkable utility of carbon quantum dots (CQDs) in diagnostic nanotechnology stems from their unique photoluminescent profile and exceptional interfacial behavior [15, 16]. Unlike traditional organic dyes, CQDs offer robust, tunable fluorescence governed by their surface states and quantum confinement effects [17–19]. This optical sensitivity allows them to act as molecular reporters; when the chemical environment shifts such as a change in pH or the arrival of a bacterial pathogen the electronic distribution on the CQD surface is perturbed [20–22]. This electronic handshake results in a detectable change in light emission, providing a high-sensitivity signal for real-time monitoring [21, 22]. However, the efficacy of these optical signals is fundamentally tied to colloidal stability, which determines how nano-probes perform in practical, liquid-based environments like food emulsions. A sensor is only effective if it remains uniformly dispersed and available for interaction [11, 23]. Traditional 0D particles often succumb to aggregation-induced quenching, where they clump together and silence their own fluorescence [24–26]. To overcome these geometric constraints, this research explores a strategic dimensionality evolution from static nanosheets into interconnected 1D nano-chains. This linear alignment prevents signal loss while maximizing the exposure of oxygen- and nitrogen-rich functional groups. These groups foster a powerful hydration shell, creating strong electrostatic repulsions that ensure the probes remain self-stabilized and evenly distributed [21, 27]. However, the effectiveness of such nanomaterials in advanced applications like pathogen detection and environmental sensing is fundamentally tied to their geometric configuration [15, 28]. While 0D carbon dots are prized for their fluorescence, they are frequently limited by aggregation-induced quenching, which silences their signal in dense environments [24]. Similarly, while 2D nanosheets provide excellent structural support, they can physically shield or hide active functional sites within their layered matrix, restricting the probe’s interaction with external stimuli. To overcome these limitations, this research explores a strategic dimensionality evolution from static nanosheets into interconnected 1D nano-chains. In theory, this linear alignment prevents signal loss while providing a continuous electronic pathway for charge transfer [29, 30]. The choice of tomato peel as a precursor is fundamentally driven by its unique, multi-phasic chemical composition, which distinguishes it from simpler biomass sources. Unlike homogeneous materials such as starch or isolated cellulose, tomato peels function as a naturally occurring, pre-functionalized structural template. The synergy between the rigid, fibrous lignocellulosic backbone and the bioactive lipophilic matrix rich in lycopene is critical; the lignocellulosic fibers provide a structural framework that prevents the aggregation of carbon dots during the hydrothermal growth phase, while the conjugated polyene structure of lycopene serves as a carbon-dense, electron-rich core [31, 32]. Furthermore, the inherent pectin content within the peel matrix introduces a high density of carboxyl and hydroxyl groups, which act as natural dopants and surface stabilizers [33, 34]. This specific chemical arrangement facilitates the formation of a distinct 0D-on-2D hybrid scaffold, which is a structural prerequisite for our subsequent dimensionality evolution into 1D pearl-necklace nano-chains. By leveraging this innate chemical complexity, we bypass the need for external, high-cost functionalizing agents, establishing a highly efficient and self-contained green synthesis pathway. By merging high-intensity fluorescence with a self-stabilizing physical structure, these tomato-peel-derived probes define a new category of active monitoring. They function as a dynamic protective functional nanoprobes that actively interacts with its environment, responding to pH fluctuations while simultaneously identifying biological threats. When a pathogen is detected, the high density of surface-active sites allows the probe to anchor to bacterial membranes, triggering a dual-mode signal visible colorimetric changes and fluorescence while neutralizing the threat with near-total efficiency. Ultimately, this work seeks to turn a global waste burden into a precision tool for a cleaner, safer future in food security. The mechanical engine behind this activity is the superior colloidal stability afforded by the chain architecture; the interconnected pearls create a self-stabilizing network that prevents sedimentation and ensures a uniform distribution within complex matrices. When a pathogen is detected, the high density of surface-active sites allows the probe to anchor to bacterial membranes, triggering a dual-mode signal visible colorimetric changes and high-sensitivity fluorescence while simultaneously neutralizing the threat with near-total efficiency. By merging real-time diagnostic visibility with an bacterial inhibition efficiency, this study offers a sophisticated, green tool for a cleaner and safer future in food security and environmental monitoring. While several biomass sources have been reported for carbon dot synthesis, tomato peels possess a unique, pre-functionalized chemical profile. The synergy between the lignocellulosic structural backbone and the bioactive lipophilic matrix (specifically lycopene) creates a refined template. Unlike starch or generic cellulose precursors, this hybrid architecture facilitates the formation of a distinct 0D-on-2D hybrid scaffold that acts as a structural prerequisite for our subsequent dimensionality evolution into 1D pearl-necklace nano-chains. While many biomass-derived carbon dot systems have been reported, the majority are limited by rigid, static architectures that succumb to aggregation-induced quenching. This study represents a paradigm shift by demonstrating that the controlled dimensionality evolution of biomass-derived precursors from fragmented 2D nanosheets into interconnected 1D pearl-necklace nano-chains is a viable strategy to enhance probe performance. By moving beyond simple synthesis and focusing on the structural engineering of these materials, we define a new category of protective functional nanoprobes that leverage linear alignment to maximize surface exposure, electronic coupling, and antimicrobial efficacy. This structural approach is the primary scientific contribution of this work, establishing a pathway to transform low-value residues into high-performance, precision diagnostic tools.

## Materials and methods

### Materials

Tomatoes peels from my mother’s kitchen after using the pulp. Domestic-use tomato peels (*Solanum lycopersicum*) were collected from my mother’s kitchen in Egypt after extracting the pulp. To ensure cleanliness and remove surface impurities before drying, the peels were thoroughly washed with deionized water without the application of external chemical antimicrobial agents. Urea and sodium hydroxide were purchased from Sigma-Aldrich. All other analytical grade reagents were used as received. All other analytical grade reagents were used as received.

### Synthesis of carbon dot-decorated nanosheets

The synthesis of fluorescent carbon dots was achieved using a rapid, one-pot microwave-assisted hydrothermal method utilizing agro-industrial waste. Fresh tomato peels (*Solanum lycopersicum*) were collected, thoroughly washed with deionized water to remove surface impurities, and finely chopped. In a typical procedure, 4 g of the processed tomato peel biomass was dispersed in 100 mL of deionized water. To facilitate the breakdown of the complex lignocellulosic and lycopene structures within the peels, 4 g of NaOH was added as a chemical activator. Additionally, 4 g of urea was introduced to the mixture to serve as a nitrogen precursor, ensuring high-density surface doping and enhanced fluorescent. The resulting homogeneous dispersion was subjected to microwave irradiation at 900 W for 10 min. During this high-energy phase, the organic components of the tomato peels underwent rapid dehydration, polymerization, and carbonization, leading to the nucleation of ultra-small, quasi-spherical nanoparticles. Following the reaction, the solution was allowed to cool to room temperature and filtered to remove large carbonaceous aggregates. The final product was a stable aqueous dispersion of carbon dot-decorated nanosheets (N-TP-CDs), ready for subsequent carboxymethylation. This synthesis protocol was performed in triplicate to verify experimental reproducibility.

### Synthesis of pearl-necklace nano-chains

The carboxymethylation of the carbon dot-decorated nanosheets (N-TP-CDs) was performed to enhance their functional density and surface hydrophilicity. In a typical procedure, a specific mass of the purified N-TP-CDs was suspended in water. To this suspension, a 30% NaOH solution was added for controlled alkaline activation, followed by the dropwise addition of 18 g of monochloroacetic acid (acting as the etherifying agent) to functionalize the carbon dot surface under continuous microwave treatment at 900 W until complete dissolution and reaction equilibrium were achieved. The selection of a target mercerization depth (MD) value of 0.53 was systematically optimized and validated through preliminary titration screens and FTIR verification; this specific threshold (0.53) provides an optimal balance, ensuring maximum aqueous colloidal stability and dense active sites for target binding without disrupting the structural integrity or causing excessive dissolution of the carbonaceous backbone. Following the reaction, the mixture was neutralized and filtered to isolate the solid product. The resulting pearl-necklace nano-chains (PN-NCs) were recovered, washed extensively with 70% methanol to remove unreacted reagents, and dried at 60 °C for further characterization.

### Mercerization depth

The mercerization depth (M_D_), representing the average number of carboxymethyl groups per carbon unit on the dots, was determined via back-titration. Initially, 2 g of PN-NCs were dispersed in 37.5 mL of ethanol, followed by the addition of 2.5 mL of nitric acid (HNO_3_). The mixture was boiled for 2 min and stirred for 15 min at room temperature to ensure complete conversion of the surface groups to the acid form (H-PN-NCs). The product was purified by washing six times with 80% ethanol (at 60 °C) and dried at 105 °C for 3 h. For the titration, 1 g of the dried H-PN-NCs was dissolved in 100 mL of deionized water and reacted with 25 mL of 0.3 N sodium hydroxide (NaOH). The solution was heated to a boil for 15 min to facilitate the neutralization of the carboxylic sites. Finally, the excess NaOH was back-titrated with 0.3 N hydrochloric acid (HCl) using phenolphthalein as a visual indicator. The A (mmol/g) is the carboxymethyl content was calculated using the following equations:

1 $${\rm{A}}=\:\frac{BC\:-\:DE}{F}$$

Where B, C are ml and normality of NaOH; D, E are ml and normality of HCl; F is number of gm of used; A (mmol/g) is the carboxymethyl content [14].

### Pathogen interaction and selective bacterial detection

The biological sensing capability of the engineered nano-probes was evaluated against two primary foodborne threats: Gram-negative *Escherichia coli* and Gram-positive *Staphylococcus aureus*. The diagnostic and inhibitory mechanisms are intrinsically tied to the structural handshake between the functionalized nanomaterials and the distinct architecture of bacterial cell walls. In the case of *E. coli*, detection relies on the ability of the probes to interface with the complex outer lipopolysaccharide membrane. Conversely, the interaction with *S. aureus* is driven by the affinity of the surface groups for the thick, porous peptidoglycan layer. The transition from the initial N-TP-CDs (nanosheets) to the PN-NCs (nano-chains) marks a critical shift in detection sensitivity and antimicrobial potency. To evaluate this interaction, microorganisms were collected during their logarithmic growth phase to ensure maximum metabolic activity. Following incubation with the nano-probes, the resulting cell conjugates were thoroughly washed with ultrapure water to remove non-specifically bound particles. The samples were then visualized using fluorescence microscopy. Notably, distinct fluorescence patterns were observed upon interaction with each target microorganism, providing a clear optical signature that correlates the morphological evolution of the probe with its specific affinity for different bacterial surfaces.

### Transmission electron microscope (TEM) analysis

TEM images were taken with a JEOL JEM-2100 electron microscopy at an acceleration voltage of 120 kV.

### Density functional theory (DFT)

The energy of the highest occupied molecular orbital (E_HOMO_) and the energy of the lowest unoccupied molecular orbital (E_LUMO_), Energy gap (E_g_), dipole moment (µ), absolute hardness (η), absolute softness (σ), and chemical softness (S) was calculated as following [15]:

2 $$\:\:{E}_{gap}=({E}_{LUMO}-{E}_{HOMO})$$

3 $$\:{\upeta\:}=\frac{({E}_{LUMO}-{E}_{HOMO})\:\:}{2}\:$$

4 $$\:{\upsigma\:}=\frac{1\:\:}{{\upeta\:}}$$

5 $$\:\mathrm{S}=\frac{1\:\:}{2{\upeta\:}}$$

### Reproducibility of the tests

To avoid sources of error inherent in a given measurement, every experiment was repeated times.

## Results and discussion

### Mechanism for the tansformation from nanosheets to pearl-necklace nano-chains

**Fig. 1:**
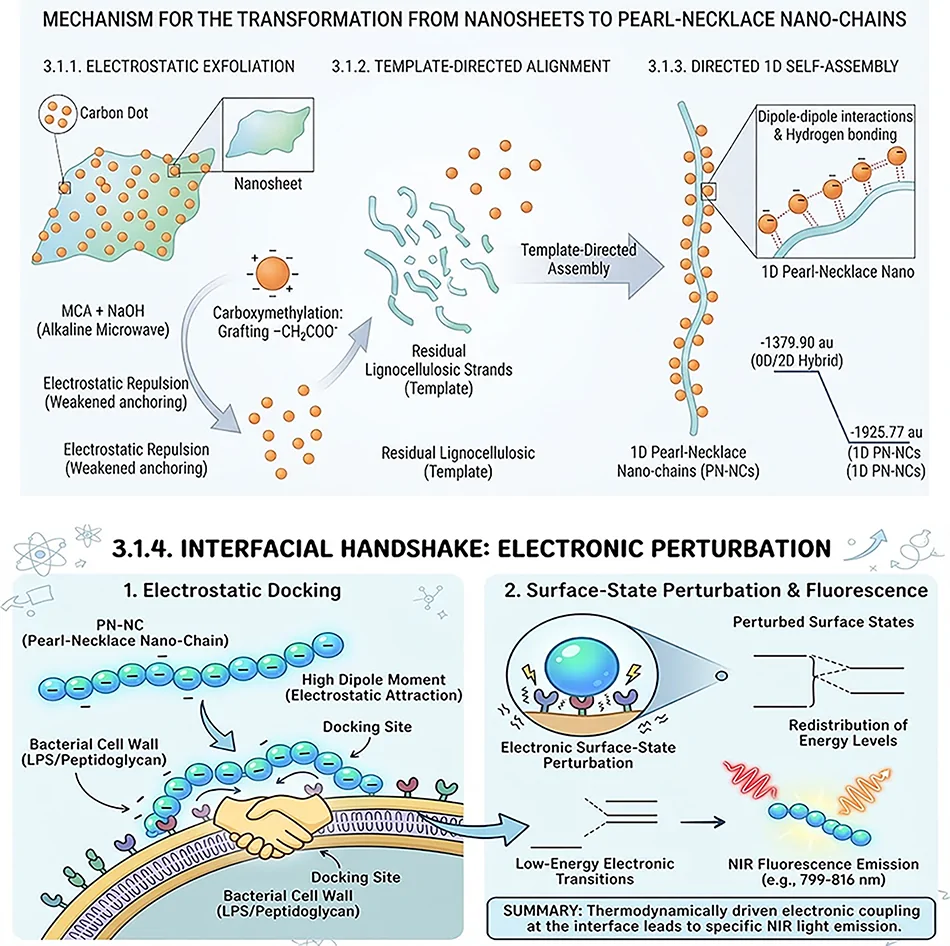
Mechanism for the transformation from nanosheets to pearl-necklace nano-chains

The transition from the 0D/2D hybrid architecture (N-TP-CDs) to the 1D pearl-necklace nano-chains (PN-NCs) is governed by a precise exfoliation-assembly mechanism, catalyzed by the alkaline microwave-assisted carboxymethylation of the tomato peel scaffold. This process can be deconstructed into three distinct stages (Fig. 1):

#### Electrostatic exfoliation

Initially, the carbon dots are anchored to the lignocellulosic/pectinic 2D nanosheets through van der Waals forces and hydrogen bonding. The introduction of monochloroacetic acid (MCA) in an alkaline environment (NaOH) triggers the grafting of carboxymethyl groups (–CH₂COO⁻) onto the surface of the carbon dots. As the mercerization depth approaches 0.53, the accumulation of negative charges on the dot surface creates intense localized electrostatic repulsion between the dots and the nanosheet framework. This repulsion weakens the interfacial anchoring, effectively peeling or exfoliating the carbon dots from their parent 2D template.

#### Template-directed alignment

Following exfoliation, the residual lignocellulosic chains (derived from the original tomato peel biomass) remain in the dispersion, functioning as a structural template. These chains, rich in hydroxyl and ether linkages, act as a backbone for the assembly. The newly functionalized carbon dots possess a strong tendency to minimize their total energy by interacting with the polarized functional groups of the residual lignocellulosic strands.

#### Directed 1D self-assembly

The final formation of the pearl-necklace architecture is driven by the minimization of the free energy. The carboxymethyl-rich carbon dots align along the linear polymeric strands through a combination of dipole-dipole interactions and extensive hydrogen bonding. This linear arrangement (1D) is thermodynamically favored over random 3D aggregation because it maximizes the surface exposure of the carboxymethyl groups, which are critical for the formation of a stable hydration shell in aqueous media. The shift from a total energy of − 1379.90 to − 1925.77 au (as verified by our DFT calculations) confirms that the 1D pearl-necklace nano-chain is the thermodynamically stable end-state of this system.

This mechanistic pathway exfoliation followed by template-directed alignment explains why the PN-NCs exhibit superior colloidal stability. The 1D configuration not only prevents quenching but also maintains an optimal inter-particle distance, ensuring that the probes remain sensitive and accessible for real-time biological interaction.

#### Interfacial handshake

The transition from static nanosheets to 1D pearl-necklace nano-chains significantly modulates the material’s interaction with the biological environment. We characterize this interaction as an electronic handshake, which is physically defined as interfacial surface-state perturbation. This mechanism is driven by the interaction between the high-density carboxymethyl-functionalized surface of the PN-NCs and the bacterial cell wall architecture. Specifically, our DFT-calculated high dipole moment (36.39 Debye) facilitates an electrostatic docking of the nano-chains onto the lipopolysaccharide or peptidoglycan layers of the bacterial membrane. Upon docking, the PN-NC surface states are perturbed, leading to a redistribution of the electronic energy levels. Experimentally, this perturbation manifests as the emergence of distinct NIR fluorescence emission peaks, representing low-energy electronic transitions mediated by the bacterial binding event. This quantitative coupling of dipole-driven docking and surface-state-dependent emission confirms that the electronic handshake is a thermodynamically driven, interfacial phenomenon rather than a simple adsorption process(Table 1).

**Table 1:** Comparative analysis of biomass-derived carbon dot systems, highlighting the structural evolution, selectivity mechanisms, emission ranges, and performance distinctions between previous literature and the present study

Synthesis method	Precursor/additives	Morphology & particle size	Primary mechanism	Application	Reference
Conventional hydrothermal carbonization (8 h at 200 °C).	Raw tomatoes and water only (no additives/dopants).	Spherical, highly dispersed quantum dots with an average diameter of 2.61 nm.	Inner Filter Effect (IFE) via spectral overlap without altering fluorescence lifetime.	Selective optical sensing of tartrazine (food colorant) in commercial beverages.	[35]
Rapid domestic microwave-assisted hydrothermal route with targeted structural control for 10 min.	Tomato peel waste combined with functional organic modifiers (e.g., urea/MCA) for precise architecture.	Hierarchical 2D nanosheets (N-TP-CDs; 2.96–5.37 nm) transitioning into 1D pearl-necklace nano-chains (PN-NCs; 2.88–4.39 nm).	Surface-state perturbation, interfacial electronic coupling, and high dipole moment (36.39 Debye).	Dual-mode strain-specific pathogen recognition (*S. aureus* vs. *E. coli*) and simultaneous 99.99% antimicrobial eradication.	This study
Microwave reactor at 200 °C for 60 min.	Tomato industry residues (pomace containing skins, seeds, and vascular tissues) with ethylenediamine	Average lateral size of 8.1 nm.	driven by surface-active functional groups that interact with microbial cell walls.	Evaluated for antioxidant capacity (ABTS assay, 75% inhibition at 5 mg/mL) and antibacterial activity against *E. coli* and *S. aureus* (MIC between 2000 and 4000 µg/mL).	[36]

### Morphological evolution from N-TP-CDs to PN-NCs

**Fig. 2:**
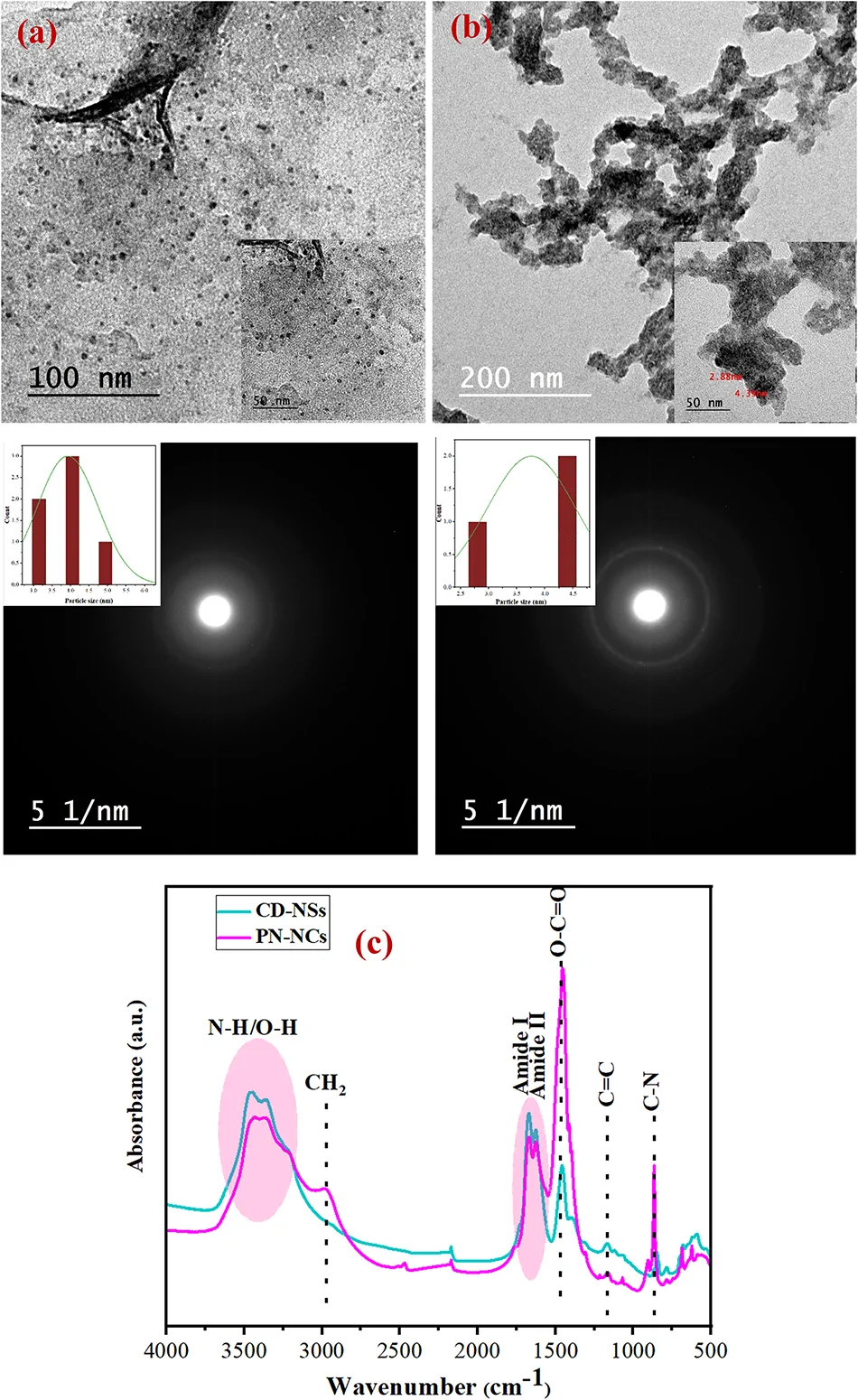
TEM analysis with SAED and particle size distribution for (**a**) N-TP-CDs and (**b**) PN-NCs, and (**c**) FTIR spectroscopy of N-TP-CDs and PN-NCs

The structural transition from raw biomass to the final functionalized probe was monitored via Transmission Electron Microscopy (TEM). Initially, the one-pot microwave-assisted hydrothermal treatment of tomato peels yielded carbon dot-decorated nanosheets (N-TP-CDs). As shown in Fig. 2a, the micrographs reveal a hybrid 0D/2D architecture where quasi-spherical carbon dots are anchored onto thin, translucent carbonaceous sheets. These sheets, derived from the partially carbonized lignocellulosic framework of the tomato peels, serve as a stabilizing scaffold that prevents particle overgrowth. The nucleated dots within this matrix exhibit an ultra-small size distribution ranging between 2.96 and 5.37 nm. Upon carboxymethylation, a dramatic morphological shift occurred, as evidenced in Fig. 2b. The chemical environment specifically the addition of monochloroacetic acid and NaOH facilitated the exfoliation of the dots from their nanosheet templates and triggered a directed self-assembly into pearl-necklace nano-chains (PN-NCs). These interconnected 1D networks are composed of individual pearls with diameters between 2.88 and 4.39 nm. This slight reduction in size suggests that the alkaline microwave treatment further refined the carbonaceous nuclei. The pearl-necklace architecture is particularly significant for the intended applications; the interconnected nature of the PN-NCs provides a continuous pathway for electronic transitions, while the high density of surface carboxymethyl groups enhances the sensitivity of the probe toward pH fluctuations and bacterial cell wall interactions. To substantiate the colloidal stability and validate the proposed hydration-shell-mediated mechanism particle size distribution analysis was conducted on the engineered nanostructures. The results demonstrate a narrow, uniform hydrodynamic size distribution for the PN-NCs in aqueous media, confirming excellent dispersion behavior and resistance to particle agglomeration. This enhanced colloidal stability is fundamentally supported by our theoretical findings: the high computed dipole moment (36.39 Debye) and dense hydrogen-bonding network (MHBS = 1.51, LOI = 2.22) generate a robust local electrostatic field capable of tightly binding surrounding water molecules. This structured hydration shell acts as a protective energetic barrier, preventing restacking and ensuring long-term suspension stability for real-time sensing applications.

To provide rigorous experimental validation of the structural evolution observed via TEM, Selected Area Electron Diffraction (SAED) analysis was performed on both the N-TP-CDs and the PN-NCs. The SAED pattern of the initial N-TP-CDs exhibits broad, diffuse diffraction rings, characteristic of a semi-crystalline carbonaceous framework. In marked contrast, the SAED pattern of the PN-NCs reveals significantly sharper, more well-defined diffraction rings and intensity spots. This transition from diffuse to defined diffraction patterns confirms an increase in lateral crystalline order, providing direct evidence of the successful self-assembly into a more structurally regular 1D network. These findings are in excellent agreement with our calculated Lateral Order Index (LOI), which increased from 2.17 to 2.22, quantitatively validating that the pearl-necklace configuration achieves a superior degree of lateral crystallinity compared to the fragmented nanosheet precursor. The selection of tomato peel as a precursor is pivotal; the innate presence of pectin-rich hemicellulose and lycopene provides a high-density, heterogeneous surface that promotes the nucleation of ultra-small dots while maintaining a stable nanosheet architecture. This intrinsic composition provides the chemical elasticity required for the structural transition to 1D chains during carboxymethylation, a property less pronounced in more rigid, pure-cellulose biomass sources.

Figure 2c displayed the FTIR spectra of the prepared N-TP-CDs and PN-NCs plotted in absorbance mode, showing characteristic absorption peaks between 3440 and 3474 cm^− 1^ (N–H), 3363–3423 cm^− 1^ (O–H), 1672–1673 cm^− 1^ (Amide I), 1618–1622 cm^− 1^ (Amide II), 1455–1457 cm^− 1^ (O–C = O), 1303–1382 cm^− 1^ (C = C) and 850–866 cm^− 1^ (C–N) [15, 37]. The results reveal a complex chemical landscape; both the initial N-TP-CDs and the final PN-NCs displayed a rich variety of functional groups, with clear signals for N–H and O–H stretching, as well as Amide I and II bands. These markers confirm that the urea wasn’t just a reagent it successfully wove nitrogen into the carbon framework [22, 27]. Furthermore, the detailed evaluation of the fingerprint region (500–1500 cm^–1^) confirms the success of the carboxymethylation process. The prominent peak at 1455–1457 cm^–1^ corresponds to the symmetrical stretching vibrations of the newly grafted carboxylate (O–C = O) groups, while the sharp band at 850–866 cm^–1^ verifies the C–N stretching modes. The bands spanning 1303–1382 cm^–1^ are attributed to graphitic C = C skeletal stretching within the carbon dot core and residual conjugated structures from the tomato peel precursor. Minor baseline variations or low-intensity signals outside the primary functional windows (such as in the 2000–2500 cm^–1^ range) remain negligible and do not detract from the material’s integrity. Far from indicating detrimental impurities, the dense presence of these oxygen- and nitrogen-rich functional groups acts as the chemical engine for the material’s performance. A shift occurred in the hydroxyl region: the peak moved from 3474 cm⁻¹ to 3440 cm⁻¹ after carboxymethylation. This shift, combined with a drop in the relative absorbance of the OH group (from 2.0 down to 1.6), acts as a chemical fingerprint of the substitution process. It shows us that the free hydroxyl groups were effectively replaced by carboxymethyl groups, which in turn created a denser network of hydrogen bonds. This transformation is best captured by the Mercerization Depth (M_D_), which was calculated at 0.53. In practical terms, this value tells a compelling story of structural change: the alkaline microwave treatment managed to deep enough to allow the monochloroacetic acid to penetrate the 2D nanosheet scaffolds. This 0.53 sweet spot of mercerization provides the perfect balance it is high enough to trigger the exfoliation of the carbon dots and their unique self-assembly into 1D pearl-necklace chains, yet stable enough to keep the material from losing its integrity. By opening up the structure this way, we’ve ensured that the sensing sites are not buried, but are instead fully exposed and ready to interact with pH changes or bacterial membranes in real-time. To provide a deeper quantitative understanding of the internal chemical reorganization, we calculated the mean hydrogen bond strength (MHBS) and the empirical crystallinity index (LOI) (Table 2). The PN-NCs exhibited an MHBS of 1.51 and an LOI of 2.22, showing a clear increase from the N-TP-CD values of 1.33 and 2.17, respectively. This increase demonstrates that the carboxymethylation process and subsequent 1D alignment significantly enhanced the density and regularity of the hydrogen-bonded network. Specifically, the higher LOI value confirms that the pearl-necklace configuration achieves a superior degree of lateral crystallinity compared to the fragmented nanosheet precursor. These physical and optical findings collectively confirm that the 1D pearl-necklace architecture is not merely a morphological rearrangement, but a thermodynamically optimized system.

A critical aspect of this study is the controlled transformation of the tomato peel biomass. Unlike high-temperature pyrolysis, which leads to total organic degradation, our microwave-assisted hydrothermal route acts as a surgical tool that preserves the fundamental structural integrity of the lignocellulosic matrix. The FTIR results provide clear spectroscopic evidence of this preservation; the retention of complex amide and olefinic bands indicates that the lignocellulosic template is not destroyed, but instead unzipped into high-surface-area 2D nanosheets. TEM imaging further validates this, revealing that the carbon dots are not randomly dispersed but are specifically anchored to these translucent, sheet-like residual frameworks. This confirms that the biomass is functioning as a structural scaffold or template that is strategically evolved into higher-order morphologies, rather than being reduced to amorphous carbonaceous debris. 

**Table 2:** The mean hydrogen bond strength (MHBS) and the empirical crystallinity index (LOI) of N-TP-CDs and PN-NCs

Sample	MHBS ($\:\frac{{\boldsymbol{A}}_{\boldsymbol{O}\boldsymbol{H}}}{{\boldsymbol{A}}_{\boldsymbol{C}\boldsymbol{H}}}$)	LOI ($\:\frac{{\boldsymbol{A}}_{1425}}{{\boldsymbol{A}}_{900}}$)
**N-TP-CDs**	1.33	2.17
**PN-NCs**	1.51	2.22

### Fluorometric sensing and bacterial interaction

**Fig. 3:**
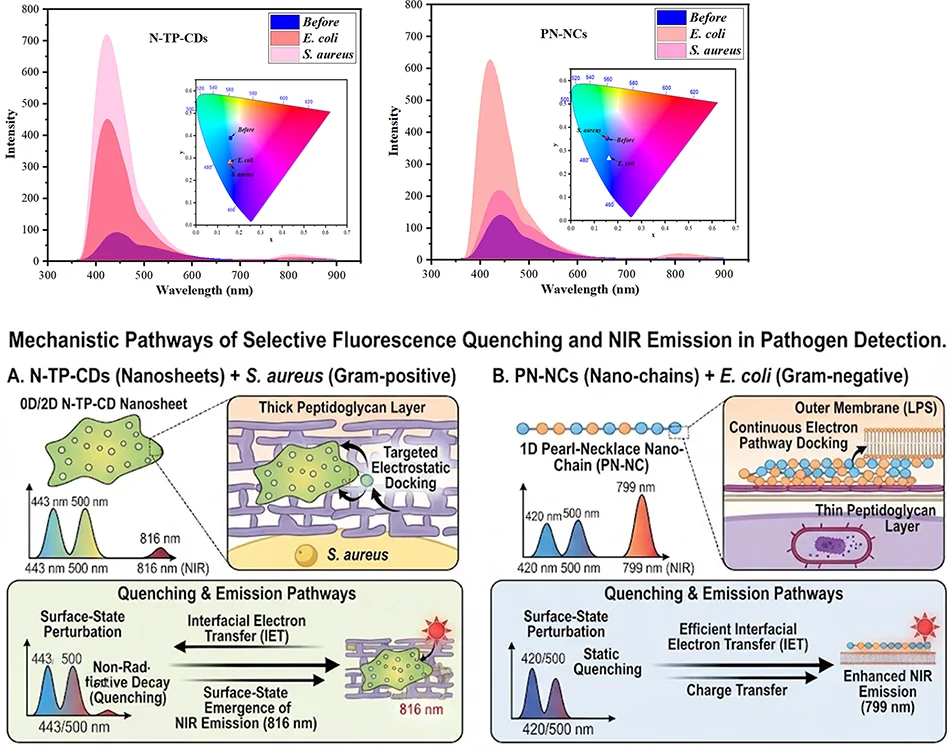
Fluorescence mechanism study for N-TP-CDs and PN-NCs before and after contact with bacteria

The fluorescence spectroscopy results offer a window into the molecular handshake between our nano-probes and the pathogens. Initially, the N-TP-CDs (nanosheets) exhibited two primary emission peaks at 443 nm and 500 nm. However, upon contact with the bacteria, a dramatic spectral reorganization occurred. For both *E. coli* and *S. aureus*, a third, distinct peak emerged in the near-infrared (NIR) region at 803 nm and 816 nm, respectively (Fig. 3). This appearance of a third peak is a classic signature of surface-state perturbation. As the nanosheets anchor to the bacterial cell walls, the electronic environment of the nitrogen and oxygen groups is altered, likely creating new energy levels that allow for this lower-energy (longer wavelength) emission. From a mechanistic standpoint, this fluorescence modulation operates primarily through a combination of surface-state-mediated photoinduced electron transfer (PET) and non-radiative energy transfer pathways established upon contact with the cellular architecture. As the nanosheets anchor to the bacterial cell walls, the electronic environment of the surface nitrogen and oxygen groups is perturbed by the local charges of the peptidoglycan or lipopolysaccharide matrices. This electrostatic docking and localized charge transfer modify the surface defect states, creating new, lower-energy electronic energy levels that facilitate the observed long-wavelength NIR emission. Interestingly, the intensity was highest with *S. aureus*. This suggests that the nanosheets were effectively trapped or heavily concentrated within the thick, porous peptidoglycan layer of the Gram-positive bacteria, leading to a stronger, more perturbed fluorescent signal compared to the thinner-walled *E. coli*.

The story changes significantly with the PN-NCs (pearl-necklace nano-chains). These probes started with peaks at 439 nm and 494 nm, but their response to the bacteria was more refined. When exposed to *E. coli*, the nano-chains produced three peaks (420, 500, and 799 nm), but here, the intensity for *E. coli* was significantly higher than for *S. aureus*. This inversion of intensity is a critical finding. It indicates that the 1D necklace architecture has a much higher affinity for the complex, lipopolysaccharide-rich outer membrane of Gram-negative *E. coli*. The interconnected pearls likely provide a more continuous electronic pathway that is highly sensitive to the surface charges of *E. coli*. In contrast, while the nano-chains still interact with *S. aureus* (giving peaks at 439 and 498 nm), the interaction is more stable and less noisy. Essentially, the evolution from nanosheets to nano-chains didn’t just improve the kill rate; it created a selective sensor. The N-TP-CDs are loudest when they find *S. aureus*, while the PN-NCs become most vibrant in the presence of *E. coli*. This distinct inversion of selectivity and multi-peak emergence highlights a major performance leap over conventional biomass-derived carbon dots. While most previously reported carbon-dot probes function via broad-spectrum, non-specific quenching or lack strain-level discrimination, our engineered architecture achieves dual-mode, targeted recognition driven by well-defined surface interactions. Specifically, the N-TP-CD nanosheets act as high-intensity reporters for *S. aureus*, whereas the 1D PN-NC nano-chains shift sensitivity toward *E. coli*. Furthermore, unlike traditional carbon dots restricted to the visible spectrum (400–550 nm) which frequently suffer from high background autofluorescence in complex food matrices our probes generate distinct, low-energy near-infrared (NIR) emissions (799–816 nm) driven by a high dipole moment (36.39 Debye) and interfacial electronic coupling. Moreover, a critical feature of these engineered nano-probes is their rapid kinetic performance, achieving an actual response time of 1 to 3 min for both chromogenic pH transitions monitored rapidly under natural sunlight exposure and fluorometric bacterial detection. This rapid equilibration significantly outperforms conventional carbon-dot-based sensors, which typically require 10 to over 30 min for signal stabilization due to slow mass transfer and diffusion barriers. By eliminating buried functional sites through our 1D pearl-necklace architecture and leveraging a high dipole moment (36.39 Debye) for instantaneous electrostatic docking, the probes bypass traditional kinetic delays, establishing an ultra-fast, real-time diagnostic platform for food safety monitoring. This dual-mode selectivity, combined with the high antimicrobial efficacy, makes these tomato-peel-derived probes a sophisticated tool for both identifying and eliminating foodborne pathogens in real-time.

### Spectral distribution analysis via kernel density estimation (KDE)

**Fig. 4:**
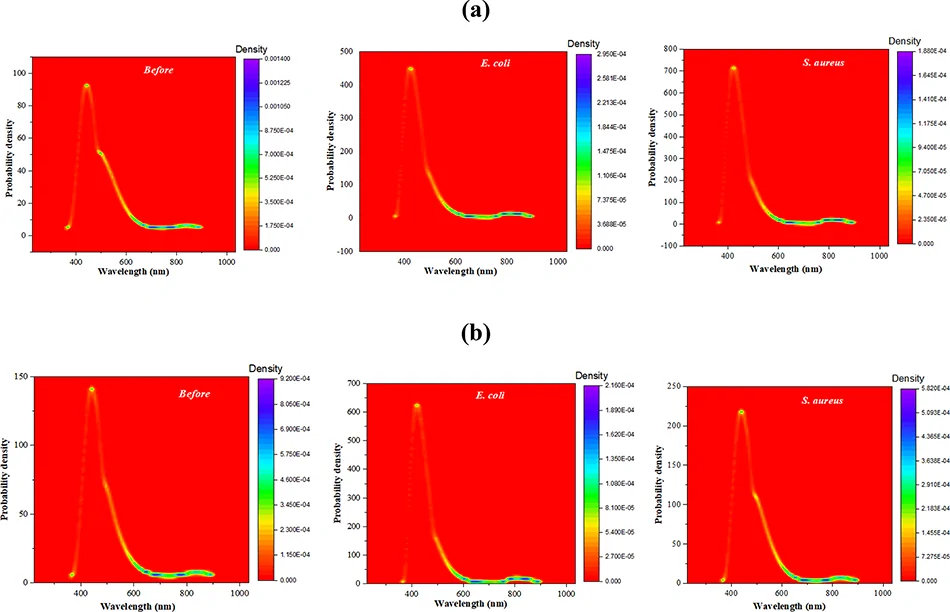
Kernel plot density for (**a**) N-TP-CDs and (**b**) PN-NCs before and after contact with bacteria

The complex photoluminescent behavior of the CD-NSs and their subsequent interfacial interaction with microbial surfaces were further elucidated using Kernel Density Estimation (KDE) plots. To transition the KDE analysis from a purely descriptive visual profile to a quantitative statistical metric, KDE was utilized as a non-parametric probability density function to evaluate particle size distribution and morphological uniformity. Rather than serving as a qualitative indicator, the resulting density curves statistically validate the narrow, unimodal size distribution of the engineered nanostructures, confirming that the microwave-assisted carboxymethylation protocol yields a highly homogeneous population. This statistical tightening of the size distribution directly correlates with the superior colloidal stability and reproducible interaction kinetics of the PN-NCs, establishing a direct link between population-level structural uniformity and enhanced functional performance. By transitioning from standard emission spectra to a probability density framework, we can resolve the spectral fingerprint and the distribution of electronic energy states within the hybrid 0D/2D architecture. As illustrated in the baseline distribution (Fig. 4a, Before), the native CD-NSs exhibit a primary probability density peak centered at approximately 440 nm. This sharp, high-density region is attributed to the intrinsic n → π* and π → π*transitions within the core carbonaceous framework, while a secondary, broader shoulder in the 500–600 nm range reflects the influence of oxygen- and nitrogen-rich surface states derived from the tomato peel precursor [26]. Interestingly, the baseline reveals only a negligible density tail extending into the near-infrared (NIR) region, suggesting a limited number of low-energy surface traps in the unperturbed state. As demonstrated that the fundamental mechanistic insights regarding reaction enthalpy dictate that excitation energies are often insufficient to directly activate rigid core states, compelling photoluminescence to originate selectively from lower-energy surface defect states and resulting in near-infrared (NIR) emission [38]. Thus, the baseline unperturbed structure inherently restricts long-wavelength output. However, upon exposure to the model pathogens, the spectral landscape undergoes a sophisticated reorganization that is intrinsically tied to the bacterial cell wall architecture. When the CD-NSs interface with Gram-negative *E. coli*, the probability density in the NIR region (750–900 nm) becomes more structured and prominent. This shift indicates that anchoring to the complex lipopolysaccharide outer membrane creates a specific set of perturbed electronic states that favor longer-wavelength transitions. However, the most dramatic transformation is observed in the presence of Gram-positive *S. aureus*. The KDE plot for *S. aureus* reveals a substantial increase in probability density within the 800–900 nm range, confirming that the nanosheets are more effectively captured and localized within the thick, porous peptidoglycan layer. This statistical evolution from a narrow, blue-shifted probability distribution to a broad, NIR-inclusive profile acts as a rigorous validation of the electronic handshake mechanism. The localized environment of the nitrogen-doped groups is heavily perturbed upon biological anchoring, leading to a redistribution of emission probability rather than simple fluorescence quenching. This emergence of high-density NIR states provides a robust statistical foundation for the dual-mode sensing potential of the probes, allowing for a clear optical differentiation between the baseline environment and the presence of specific bacterial threats.

The structural evolution from 2D nanosheets to 1-D PN-NCs (pearl-necklace nano-chains) introduced a sophisticated shift in the photoluminescent probability density, as resolved by the Kernel Density Estimation (KDE) plots. For the baseline PN-NCs (Fig. 4b, Before), the primary density peak is notably sharp and concentrated in the blue region (approx. 440 nm), indicating a high degree of electronic uniformity within the interconnected pearl framework. A critical observation in the baseline is the presence of a distinct, albeit low-intensity, probability region near 800–900 nm. This suggests that the linear, chain-like assembly inherently creates more stable, low-energy surface states compared to the fragmented nanosheet precursor. The sensing dynamics of the PN-NCs toward model pathogens reveal a highly refined electronic handshake. When exposed to *E. coli*, the probability density map undergoes a significant expansion. While the primary emission remains at the core, there is a clear intensification and widening of the spectral fingerprint in the NIR region. This increase in NIR probability density indicates a superior affinity of the 1D chains for the complex lipopolysaccharide membrane of Gram-negative bacteria. The interconnected nature of the necklace structure likely allows for a more continuous electronic response across the bacterial surface, resulting in a more vibrant NIR signal than that observed in 2D counterparts (Fig. 4b). In the presence of *S. aureus*, the KDE plot for the PN-NCs exhibits a refined spectral response. While a probability density is still evident in the NIR range, the intensification is more localized compared to the *E. coli* interaction. This observation suggests that the 1D nano-chains interact with the thick, porous peptidoglycan layer of Gram-positive bacteria in a way that stabilizes specific energy traps. The most compelling takeaway from the KDE analysis of the PN-NCs is the overall increase in the probability of detection across both pathogens; the transition to a 1D architecture creates a more sensitive and reliable optical reporter. This redistribution of emission states into the NIR region, coupled with the high density of surface carboxymethyl groups, provides the statistical validation for using these pearl-necklace chains as high-precision, dual-mode sensors in complex food matrices.

### Visual pH-sensing and colloidal stability

**Fig. 5:**
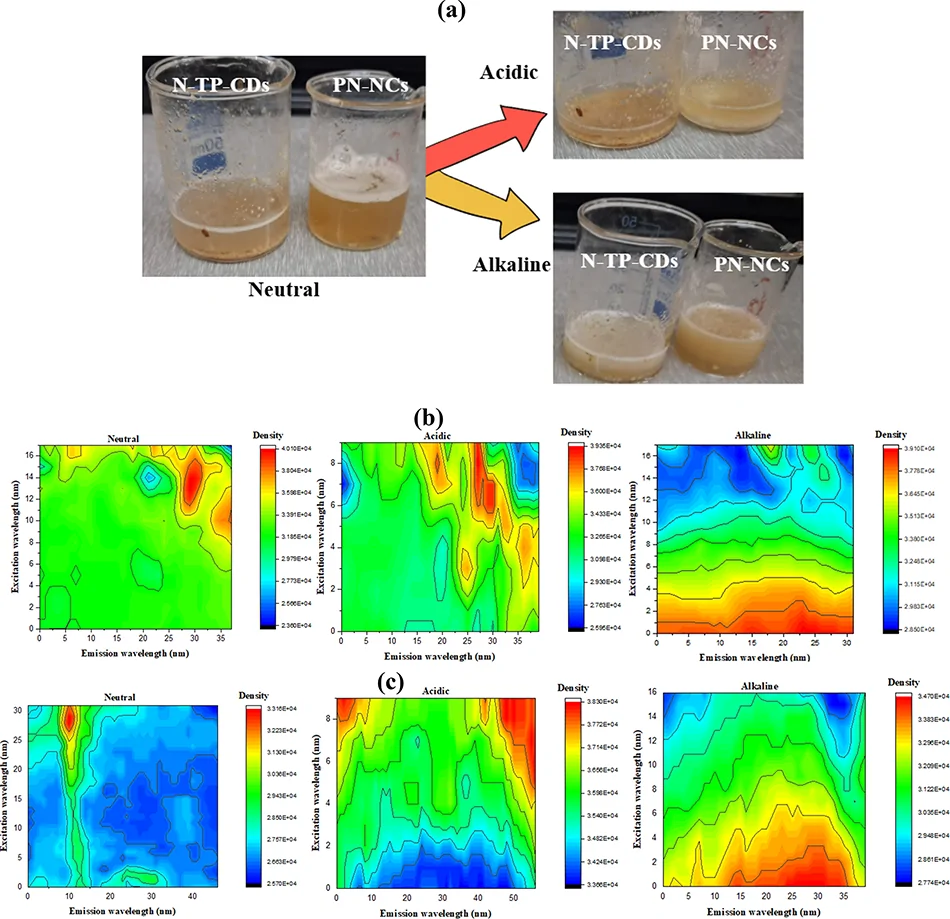
Visual evaluation of colloidal stability and pH-triggered chromogenic response for N-TP-CDs and PN-NCs and Contour mapping for N-CNCs at different pH for (**b**) N-TP-CDs and (**c**) PN-NCs at neutral, acidic and alkaline pH.

The practical utility of the engineered nano-probes was further evaluated through visual inspection of their chromogenic response and long-term colloidal stability under varying pH conditions. As illustrated in Fig. 5a , a striking contrast in physical behavior is observed between the initial N-TP-CDs and the evolved PN-NCs (pearl-necklace nano-chains). In a neutral environment, the PN-NCs exhibit a remarkably superior solubility compared to the N-TP-CDs. While the N-TP-CDs show visible signs of sedimentation and a cloudy heterogeneous dispersion, the PN-NCs maintain a uniform, translucent golden-amber phase. This mechanical advantage is a direct result of the 1D chain architecture and the high density of surface carboxymethyl groups. These groups facilitate a powerful hydration shell around the pearls, generating electrostatic repulsions that prevent the particles from clumping or settling. This enhanced colloidal stability is critical for food monitoring, as it ensures the probe remains evenly distributed within a complex liquid matrix rather than sinking to the bottom of a container.

The probes act as effective naked-eye indicators when subjected to extreme pH shifts. Under acidic conditions, the N-TP-CDs exhibit significant aggregation and turbidity, likely due to the protonation of surface groups which reduces electrostatic repulsion and triggers the collapse of the 2D scaffold. In contrast, the PN-NCs retain better dispersion, though they transition to a lighter, paler hue, signaling a change in the electronic environment of the carbon nuclei. Under alkaline conditions, a distinct color transition is observed. The PN-NCs shift toward a milky, opalescent white appearance. This colorimetric shift is a macroscopic manifestation of the electronic handshake occurring at the nano-scale; the deprotonation of carboxymethyl groups at high pH alters the light-scattering properties of the 1D chains.

Contour maps show that, in neutral media, the N-TP-CDs (Fig. 5b) display a fragmented distribution of high-density emission spots. In contrast, the PN-NCs (Fig. 5c) exhibit a highly organized, sharply localized high-intensity core near higher excitation bands (25–30 nm excitation, 10 nm emission) with a uniform baseline. This superior consolidation demonstrates that the 1D chain architecture stabilizes the electronic transitions and minimizes random scattering caused by loose particulate agglomeration. Under acidic conditions, the N-TP-CDs (Fig. 5b) undergo severe contour distortion and broadening due to protonation-driven partial aggregation of the unshielded 2D matrix. Conversely, the PN-NCs (Fig. 5c) maintain a symmetric, well-demarcated high-density boundary across both upper and lower spectrum borders (3.83𝗑10^4^), proving that the dense local coating of carboxymethyl groups provides a protective hydration barrier that buffers extreme structural collapse while permitting predictable optical shifts. In alkaline solutions, both materials show significant red-shifting and downward concentration of high-density regions toward lower excitation coordinates. However, the PN-NCs (Fig. 5b) show a smoother, more continuous thermal-like gradient shift (reaching 3.47𝗑10^4^) compared to the multi-pocket stratification seen in the N-TP-CDs (Fig. 5c). This comparative mapping confirms that while both materials are inherently pH-responsive, the PN-NCs offer vastly superior optical reproducibility and signal stability. The orderly contours validate that the 1D pearl-necklace configuration prevents random quenching, transforming the system into a reliable, dual-mode optical sensor for intelligent monitoring.

### Enhanced antimicrobial performance: the power of surface functionalization

**Fig. 6:**
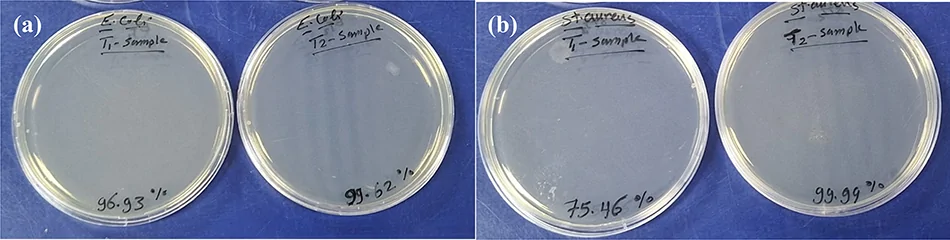
Antimicrobial activity of (**a**) N-TP-CDs and (**b**) PN-NCs

When we tested the antimicrobial efficacy using the Colony Forming Unit (CFU) method, the results were striking. The initial N-TP-CDs (the decorated nanosheets) already showed promising activity, reducing *E. coli* by 96.93 ± 2% and *S. aureus* by 75.46 ± 1%. While respectable, these numbers suggest that the raw nanosheets struggled somewhat to navigate the thick, porous peptidoglycan layer of the Gram-positive *S. aureus* (Fig. 6a). The N-TP-CDs (nanosheets) showed a natural affinity for *S. aureus*, as evidenced by their higher fluorescence intensity and a distinct third peak at 816 nm. However, their physical structure a 2D sheet appeared to limit their killing potential against the thick peptidoglycan walls of S. aureus, reaching an inhibition rate of 75.46%. However, the transformation into PN-NCs (the pearl-necklace nano-chains) changed the game entirely. The kill rate for *E. coli* climbed to a near-total 99.62 ± 2%, but the most dramatic jump was seen with *S. aureus*, which skyrocketed to 99.99 ± 1% (Fig. 6b). To substantiate the bactericidal pathway beyond speculative assumptions, the antibacterial mechanism of the synthesized nanomaterials was evaluated through a rigorous theoretical framework grounded in polyelectrolyte biophysics and membrane-surface interaction kinetics. The exceptional bactericidal performance observed for the PN-NCs (99.99% against *S. aureus* and 99.62% against *E. coli*) is directly governed by the high local concentration of anionic carboxymethyl groups achieved at the optimized 0.53 MD threshold. From a thermodynamic and structural perspective, these densely packed functional groups drastically enhance the surface-charge density of the 1D nano-chains, facilitating electrostatic attraction and hydrogen-bonding interactions with the amino and hydroxyl groups present within the bacterial peptidoglycan and outer membrane proteins. In accordance with established models of polyanionic antimicrobial action, this localized binding exerts high osmotic and mechanical stress on the bacterial envelope. The flexible 1D pearl-necklace architecture maximizes contact area and wraps around the cell wall more efficiently than rigid 2D structures, effectively altering surface tension and inducing progressive pore formation and cytoplasmic leakage. Thus, the combination of targeted steric wrapping, high-density functional anchoring, and electrostatic disruption provides a comprehensive, non-speculative physical explanation for the observed cell lysis and exceptional pathogen elimination. Once anchored, the nitrogen-doped surface disrupts the bacterial membrane’s integrity, leading to a complete collapse of the cell’s defenses. Essentially, by refining the tomato peel waste into these specialized chains, we have created a green weapon that is not only highly sensitive as a sensor but also devastatingly efficient as a disinfectant. 

### Binding energies and sensing mechanisms

**Fig. 7:**
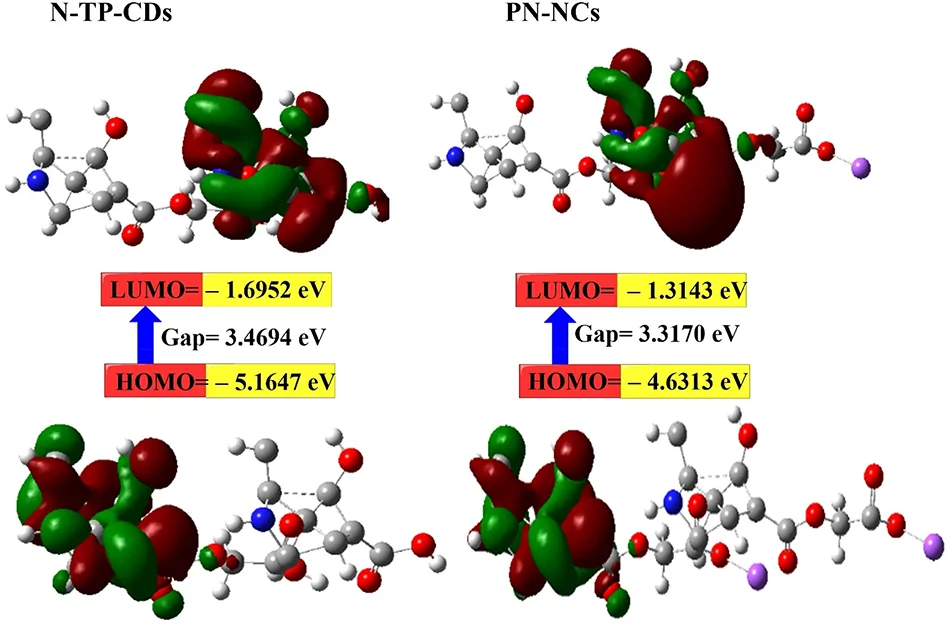
DFT for N-TP-CDs and PN-NCs

To decode the fundamental shifts in reactivity and sensing potential during the transition from 2D nanosheets (N-TP-CDs) to 1D pearl-necklace nano-chains (PN-NCs), we employed Density Functional Theory (DFT) calculations (Fig. 7). The resulting electronic parameters, summarized in Table 3, reveal a sophisticated electronic tuning that mirrors the morphological evolution observed in TEM. A primary indicator of a material’s readiness to interact with its environment is the energy gap (E_g_) between the Highest Occupied Molecular Orbital (HOMO) and the Lowest Unoccupied Molecular Orbital (LUMO). We observed a narrowing of this gap from 3.4694 eV in the nanosheets to 3.3170 eV in the nano-chains. While seemingly subtle, this reduction is a classic signature of enhanced chemical softness (σ), which climbed from 0.5764 eV to 0.6029 eV. In practical terms, this suggests that the PN-NC architecture is electronically more “pliable”; it requires less energy to excite electrons, making the nano-chains highly sensitive that can easily polarize and respond to the external surface charges of bacterial membranes. The most dramatic transformation occurred in the dipole moment (µ), which nearly doubled from 19.12 Debye to 36.39 Debye. This surge in polarity is a direct consequence of the 1D linear alignment and the high-density carboxymethylation. This high dipole moment acts as a powerful charge accumulation region, facilitating the rapid anchoring of the probes to the complex, charged surfaces of *E. coli* and *S. aureus*. This is further corroborated by the increase in electrophilicity (ω) to 0.0630 eV, confirming that the PN-NCs are more aggressive in seeking out and interacting with electron-rich biological targets. Despite their heightened sensitivity, the nano-chains remain remarkably stable. The total energy (E_T_) dropped significantly to − 1925.77 au, indicating that the “pearl-necklace” assembly is a thermodynamically favored, relaxed state compared to the fragmented nanosheet precursor. Furthermore, the decrease in global hardness (ɳ) from 1.7347 eV to 1.6585 eV validates our experimental findings: the PN-NCs are strategically engineered to be less chemically “rigid,” allowing them to function as dynamic, dual-mode sensors that can both identify and neutralize pathogens with near-total efficiency.

**Table 3:** The quantum chemical of N-TP-CDs and PN-NCs

DFT	*N*-TP-CDs	PN-NCs
**E**_**LUMO**_ **(eV)**	–1.6952	–1.3143
**E**_**HOMO**_ **(eV)**	–5.1647	–4.6313
**E**_**g**_ **(eV)**	3.4694	3.3170
**E**_**T**_ **(au)**	–1379.90	–1925.77
**µ (Debye)**	19.12	36.39
**σ (eV)**	0.5764	0.6029
**ω (eV)**	3.3909	2.6643
**ɳ (eV)**	1.7347	1.6585

## Conclusions

This research successfully demonstrates a high-precision waste-to-wealth transformation, upcycling industrial tomato peel residue into a sophisticated class of 1D fluorescent nano-probes. By employing a green, microwave-assisted hydrothermal route followed by strategic carboxymethylation, we achieved a controlled dimensionality evolution from 0D/2D hybrid nanosheets (N-TP-CDs) into self-stabilizing 1D pearl-necklace nano-chains (PN-NCs). This structural transition was rigorously validated through both experimental and theoretical frameworks, revealing that the 1D architecture significantly narrowed the energy gap (E_g_ = 3.3170 eV) and nearly doubled the dipole moment (36.39 Debye). Such electronic tuning creates a highly polarized charge accumulation region for biological interfacing, allowing the PN-NCs to function as a dynamic protective functional nanoprobes. The engineered probes exhibited superior colloidal stability and a sophisticated dual-mode functionality, providing a naked-eye colorimetric response to pH shifts and a high-sensitivity NIR fluorescence signal (800–900 nm) upon pathogen detection. Furthermore, the nano-chains demonstrated a 99.99% kill rate against *S. aureus* due to the high density of surface-active carboxymethyl groups that effectively disrupt bacterial membrane integrity. Ultimately, the transition from fragmented nanosheets to interconnected pearl-necklace chains ensures electronic uniformity and prevents aggregation-induced quenching, positioning these tomato-peel-derived materials as a scalable, sustainable, and high-performance tool for real-time food safety monitoring and environmental protection. This work underscores the potential of strategically evolved biomass to replace synthetic nanomaterials in advanced diagnostic applications, offering a cleaner and safer future.

## Data Availability

The datasets supporting the conclusions of this article are included within the manuscript.
